# Genomic Characterization and Phylogenetic Relationships of *Procypris rabaudi* Revealed by Whole-Genome Survey Analysis

**DOI:** 10.3390/ani16020246

**Published:** 2026-01-14

**Authors:** Xiaolu Han, Renhui Luo, Qi Liu, Zengbao Yuan, Wenping He

**Affiliations:** College of Fisheries, Southwest University, Chongqing 400715, China; hanxiaolu1014@163.com (X.H.); 19853507126@163.com (R.L.); liuqi_agr@163.com (Q.L.); yuanzengbao1998@163.com (Z.Y.)

**Keywords:** *Procypris rabaudi*, whole-genome survey, phylogenetic evolution, microsatellite, mitochondrial genome

## Abstract

*Procypris rabaudi*, a member of the Cyprinidae family and genus *Procypris*, has been designated as a national second-class protected wildlife species in China due to a significant decline in its wild populations. Understanding its genomic characteristics and mitochondrial genome structure is crucial for germplasm conservation and systematic classification. In this study, we utilized high-throughput sequencing to investigate the genome of *P. rabaudi*. The genome size was 1.5 Gb, with a heterozygosity rate of 0.44% and 61.47% of repetitive sequences. We identified 1,151,980 simple sequence repeats (SSRs), with mononucleotide repeats being the most abundant at 55.34%. The complete mitochondrial genome was assembled with 16,595 bp length. A phylogenetic tree constructed from 13 mitochondrial protein-coding genes indicated that genus *Procypris* was most closely related to genus *Luciocyprinus* and formed a monophyletic group with *Cyprinus*, *Carassioides*, and *Carassius*. Pairwise Sequentially Markovian Coalescent (PSMC) analysis revealed a rapid population expansion prior to the Last Interglacial Period, followed by a decline after reaching its peak during Last Glacial Period. Notably, *P. rabaudi* exhibited a two-peak demographic pattern during both the Last Glacial Period. These genomic data provide valuable resources for the conservation of *P. rabaudi* germplasm and for future studies on cyprinid classification and evolution.

## Simple Summary

*Procypris rabaudi* is an endangered cyprinid fish under national protection in China. This study performed its genome survey analysis, revealing a 1.5 Gb size with 0.44% heterozygosity and 61.47% repetitive sequences. The mitochondrial genome is 16,595 bp. Phylogenetic analysis shows *Procypris* is closest to *Luciocyprinus* and clusters with *Cyprinus* and *Carassius*. PSMC analysis indicates population expansion before the Last Interglacial, and then decline after a peak in the Last Glacial Period, featuring a unique two-peak demographic pattern. These genomic insights aid conservation and evolutionary studies of cyprinids.

## 1. Introduction

*Procypris rabaudi*, commonly known as the rock carp, a member of the family Cyprinidae, subfamily Cyprininae and genus *Procypris*, is endemic to the upper reaches and tributaries of the Jinsha River, Minjiang River, Chishui River, Jialing River and Yangtze River [1,2]. The rock carp is a benthic fish that primarily inhabits deep waters with rocky substrates; during the spawning season, it produces adhesive eggs that attach to the gravel substrate at the bottom [3]. As a typical benthic omnivore, it consumes invertebrates and organic detritus, thereby playing a pivotal role in nutrient cycling and energy transfer and contributing critically to the maintenance of biodiversity and ecosystem stability in lotic environments [2,4]. Moreover, owing to its delicate flesh and high nutritional value, *P. rabaudi* has long been an important fishery resource in its native range. However, escalating pressures from overfishing, river impoundment and pollution have precipitated a dramatic decline in wild populations [2,5]. Consequently, *P. rabaudi* is now classified as Vulnerable (VU) under China’s Red List and was elevated in 2021 to a national second-class key protected wildlife species [6]. At present, artificial breeding and restocking are the main means of protecting the rock carp. But studies on the population genetics of the rock carp based on different genetic markers have shown that the genetic diversity of the current artificially farmed rock carp is constantly decreasing [4], which may not be conducive to the protection of the rock carp. Effective conservation and sustainable utilization therefore demand robust molecular data.

The emergence and widespread adoption of next-generation sequencing (NGS) technologies have fundamentally transformed genomic studies in non-model fish species. By delivering rapid and highly cost-effective approaches to generating large-scale DNA sequence data, NGS provides unprecedented insights into genome architecture and organization, overcoming many traditional barriers in fish genomics [7,8]. As an initial critical step, genome survey sequencing allows researchers to obtain preliminary estimates of essential genomic characteristics, including overall genome size, levels of heterozygosity, and the proportion of repetitive DNA content. These surveys thereby offer a strategic foundation, informing the design and feasibility of subsequent, more comprehensive whole-genome sequencing projects [9,10]. Beyond guiding larger initiatives, genome survey sequencing itself yields immediately useful outputs. It enables the efficient assembly of complete mitochondrial genomes and facilitates the mining of simple sequence repeats (SSRs), also known as microsatellites. The polymorphic SSR markers developed through this process are invaluable tools for conducting assessments of intra-species genetic diversity, performing parentage analyses, and supporting marker-assisted breeding programs in species such as *P. rabaudi* [11,12]. Furthermore, the availability of whole-genome data empowers deeper evolutionary investigations. It allows for the reconstruction of historical population dynamics through methods like coalescent theory and demographic modeling. This provides multidimensional perspectives on a species’ evolutionary past, including signatures of population expansions, bottlenecks, migrations, and fine-scale genetic structure [13,14,15]. This integrated genomic approach has been successfully implemented across diverse teleost lineages. For instance, in species like *Acanthocepola indica* and *Tridentiger bifasciatus*, the combination of genome survey and whole-genome sequencing has been instrumental in clarifying complex phylogenetic relationships, identifying genes under selection to understand adaptive evolution, and informing conservation genetics strategies by assessing genetic health and population connectivity [16,17]. Thus, NGS serves as a cornerstone technology, enabling a multi-faceted exploration of fish genomics from fundamental characterization to applied ecological and evolutionary questions.

In the present study, we employ NGS-based genome surveying of *P. rabaudi* to: (1) characterize fundamental genomic parameters, including genome size, heterozygosity rate and repeat composition; (2) develop and validate polymorphic SSR markers for future population monitoring; (3) assemble the complete mitochondrial genome and infer phylogenetic relationships based on the 13 mitochondrial protein-coding genes (PCGs); and (4) reconstruct historical demography via the Pairwise Sequentially Markovian Coalescent (PSMC) model to detect past population size changes. The resulting genomic resources will underpin conservation management of *P. rabaudi* and enrich our understanding of cyprinid evolution and systematics.

## 2. Materials and Methods

### 2.1. Sample Collection

Adult female *P. rabaudi* (Body length: 15.4 cm, weight: 30.32 g) were obtained from an aquaculture facility in Chongqing, China. Following morphological identification, dorsal muscle tissues were excised and immediately preserved in 95% ethanol for subsequent genomic DNA extraction.

### 2.2. DNA Extraction and Sequencing

Genomic DNA was extracted from preserved muscle tissues using the classical phenol–chloroform method. The purity and concentration of DNA were assessed with a NanoDrop 2000 spectrophotometer (Thermo Fisher Scientific, Waltham, MA, USA) and a Qubit 3.0 fluorometer (Invitrogen, Carlsbad, CA, USA), respectively, by measuring A260/280 and A260/230 ratios. Integrity was confirmed through 1% agarose gel electrophoresis. High-molecular-weight DNA was sheared by sonication to an average insert size of 300–400 bp, and paired-end libraries were constructed. Sequencing was performed on the DNBSEQ platform (OneMore-Tech, Wuhan, China) and the sequencing depth was selected as 100×.

### 2.3. Raw Data Quality Control and Genome Survey

Raw reads were filtered and deduplicated using fastp v0.23.2 with default settings. The process involved the removal of low-quality reads (Phred score < 20), reads shorter than 50 bp or with excessive ambiguous bases, adapter trimming, sliding-window trimming of low-quality ends, overlap-based base correction, and PCR duplicate removal. Quality metrics (Q20, Q30, GC content) were evaluated using FASTQC v0.11.3. A 17-mer frequency distribution was computed with GCE v1.0.0 to estimate genome size, repeat content, and heterozygosity [18]. Genome ploidy was assessed using Smudgeplot v0.2.3dev with parameters “-k 21 -m 100 -ci 1 -cs 10000” [19].

### 2.4. Draft Genome Assembly and SSR Identification

High-quality reads were *de novo* assembled into contigs and scaffolds using SOAPdenovo2 v2.04 [20], the main parameters are followed the default, and “-127mer” instruction was employed to assemble the draft genome. SSRs were identified from the assembled scaffolds with the Perl script misa.pl in MISA v2.1, applying minimum repeat thresholds set at ≥10 for mono-, ≥6 for di-, and ≥5 for tri- to hexa-nucleotide motifs.

### 2.5. Mitochondrial Genome Assembly and Phylogenetic Analysis

Filtered reads were used to assemble the mitochondrial genome with MitoZ v2.4 [21], the published *P. rabaudi* mitochondrial genome (GenBank accession number: EU082030) was selected as the seed, and K-mer depth was set as 27. The annotation and visualization processes were conducted in MitoFish (version 2025.06) [22]. A phylogenetic dataset comprising 74 cyprinid species across 15 genera (the GenBank accession numbers information of all species used in this study can be found in Table S1) was compiled. To reconstruct the phylogenetic relationship of *P. rabaudi* within the Cyprinidae, we selected two outgroup species from Cobitidae (*Jinshaia sinensis*) and Catostomidae (*Thoburnia rhothoeca*), both belonging to the Cypriniformes. Considering the evolutionary rate of protein-coding genes, we employed 13 PCGs of mitochondrial genome to reconstruct the phylogenetic tree. All species’ 13 PCGs were extracted in PhyloSuite v1.2.3 [23] and aligned with MAFFT v7.471 [24]. Subsequently, the aligned PCGs were trimmed with GBlocks v0.91b [25]. The trimmed alignments were concatenated within PhyloSuite v1.2.3 [23], and the optimal substitution model was determined using ModelFinder v2.2.0 [26]. Bayesian inference (BI) was conducted in MrBayes v3.2.7a [27], which involved two independent runs, each utilizing four simultaneous Markov chain Monte Carlo (MCMC) chains. The analysis spanned 2,000,000 generations, with sampling occurring every 1000 generations. The first 25% sample data were discarded as the burn-in, and the bootstrap cycle were performed 1000 times. The resulting phylogenetic tree was visualized with iTOL v6.9: https://itol.embl.de/ (accessed on 14 October 2025).

### 2.6. Historical Demography Reconstruction

The historical dynamics of effective population size (N_e_) for *P. rabaudi* were inferred using the PSMC model. The cleaned paired-end reads of this study were aligned to the assembled reference genome [28] using BWA-MEM (-k 19 -T 30) [29]. The resulting BAM file was processed with Samtools v0.1.19 (-bF 12). The vcfutils.pl script constructs diploid FASTQ files using vcf2fq, and subsequently converts them into the psmcfa input files required for PSMC using “fq2psmcfa” script with a quality threshold of 20. The PSMC analysis was conducted under default settings (-N25 -t15 -r5 -p “4 + 25 * 2 + 4 + 6”) to infer the variation in N_e_. Finally, the “psmc_plot.pl” script was used to visualize the PSMC results with a generation time of three years, according to the mutation rate of common carp (*C. carpio*) [30], the mutation rate of *P. rabaudi* was set as “0.5 × 10^−8^”. 

## 3. Results

### 3.1. Size, Heterozygosity Ratio, and Repeat Sequence Ratio of P. rabaudi

**Figure 1:**
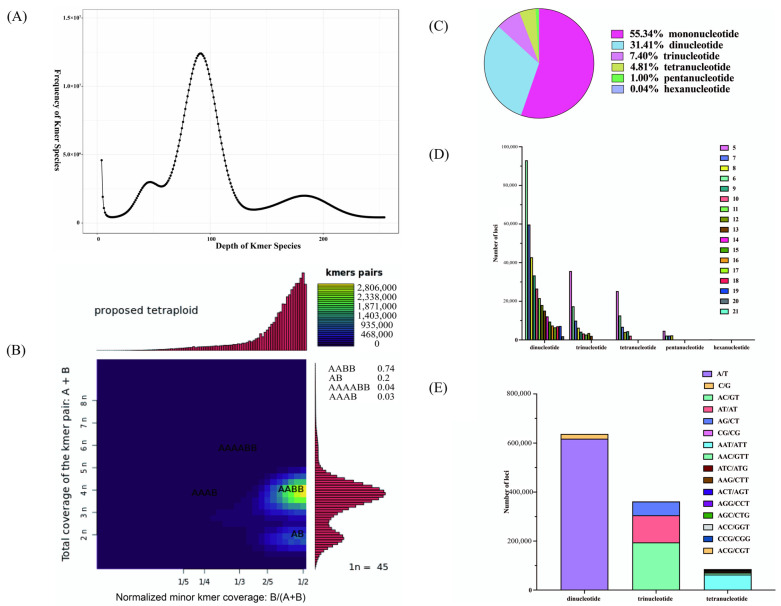
(**A**) K-mer frequency analysis of *P. rabaudi*; (**B**) Smudgeplot analysis and genotype distribution of *P. rabaudi*; (**C**) SSRs distribution of *P. rabaudi*; (**D**) The frequency of SSRs in *P. rabaudi* genome; (**E**) The motif types in *P. rabaudi* genome.

We constructed a paired-end sequencing library with an insert size of approximately 300–400 bp for *P. rabaudi* and sequenced it on the DNBSEQ platform. The raw data yielded 166.92 Gb, comprising approximately 1,112,770,540 paired-end reads (Table 1). After quality control, 157.79 Gb of clean reads were retained (approximately 1,069,509,006 reads). The clean data had Q20 and Q30 values of 99.61% and 98.49%, and GC content values of 38.91% and 38.65%, respectively, indicating the stability of the sequencing in this study (Table 1). K-mer analysis (k = 17) showed a K-mer depth of 92 for *P. rabaudi* (Figure 1A). Based on K-mer estimation, the genome size of *P. rabaudi* was approximately 1,495,420,000 bp (Table 2). The heterozygosity of the genome was estimated to be 0.44%, and the repeat sequence content was approximately 61.47% (Table 2). Furthermore, Smudgeplot analysis indicated that the AABB genotype accounted for the highest proportion at 74%, followed by the AB genotype at 20%, suggesting that the *P. rabaudi* genome is tetraploid (Figure 1B).

### 3.2. The Draft Genome Assembly and SSR Identification Results

High-quantity reads were assembled de novo using SOAPdenovo2 to generate a draft genome assembly for *P. rabaudi*. The assembly comprised 1,017,551 scaffolds, with the longest scaffold measuring 77,646 bp (Table 3). Scaffold N50 and N90 values were 7176 bp and 1292 bp, respectively. At the contig level, 4,385,755 contigs were assembled, with the longest contig spanning 10,405 bp, and the N50 and N90 contig lengths were 469 bp and 129 bp, respectively (Table 3).

The results of SSR analysis showed a total of 1,151,980 SSRs within the *P. rabaudi* genome were identified (Table 4). Among these, 227,348 sequences contained two or more SSR loci, and 156,448 SSRs were found in compound forms (Table 4). The distribution of SSR motif types was as follows: mononucleotide repeats accounted for 55.34%, dinucleotide repeats 31.41%, trinucleotide repeats 7.40%, tetranucleotide repeats 4.81%, pentanucleotide repeats 1.00%, and hexanucleotide repeats 0.04% (Figure 1C). Additionally, the frequency of SSRs decreased as the number of repeat units increased (Figure 1D). Among the motif types, A/T, AC/GT, and AAT/ATT were the most predominant for mono-, di-, and trinucleotide repeats, respectively (Figure 1E).

### 3.3. Mitochondrial Genome Assembly and RSCU Analysis

**Figure 2:**
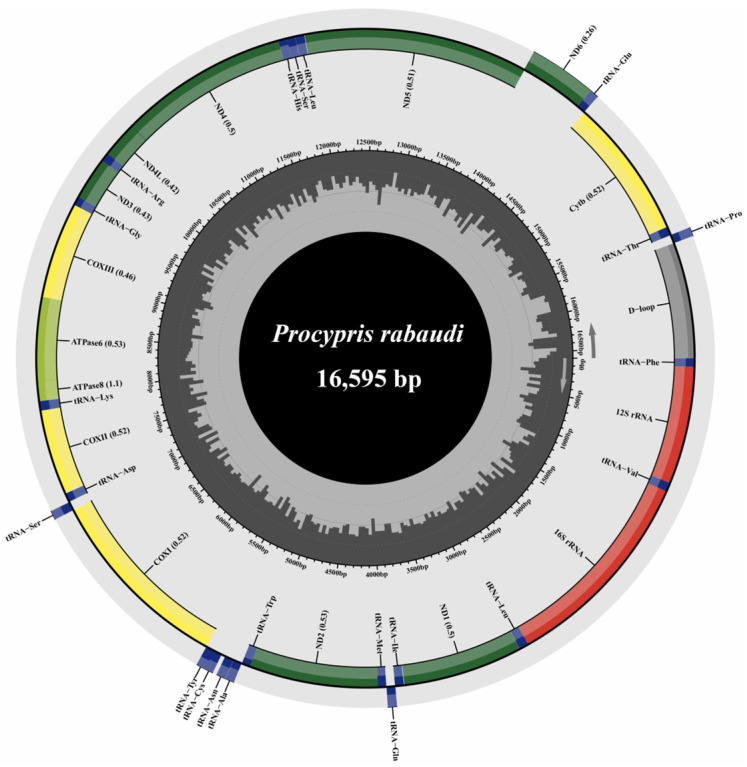
The structure of *P. rabaudi* mitochondrial genome.

The mitochondrial genome of *P. rabaudi* was successfully assembled into a closed circular molecule measuring 16,595 bp in length (Figure 2). This mitochondrial genome comprises 13 PCGs, 22 tRNA genes, two rRNA genes, and a control region. The *ND6* gene and eight tRNA genes (*tRNA-Gln*, *tRNA-Ala*, *tRNA-Asn*, *tRNA-Cys*, *tRNA-Tyr*, *tRNA-Ser*, *tRNA-Glu*, and *tRNA-Pro*) were encoded on the light strand, while the remaining 28 genes were located on the heavy strand. Regarding start codons, the *COXI* gene initiated with GTG, whereas the other 12 PCGs utilized the standard ATG start codon (Table S2). The termination codons exhibit variability: *ND1*, *COXI*, *ND4L*, *ND5*, and *ND6* utilize TAA, while *ATPase8* concludes with TAG. Additionally, *ATPase6* and *COXIII* contain incomplete stop codons (TA), whereas *ND3*, *COXII*, *ND2*, *ND4*, and *Cytb* terminate with a single T nucleotide (Table S2).

**Figure 3:**
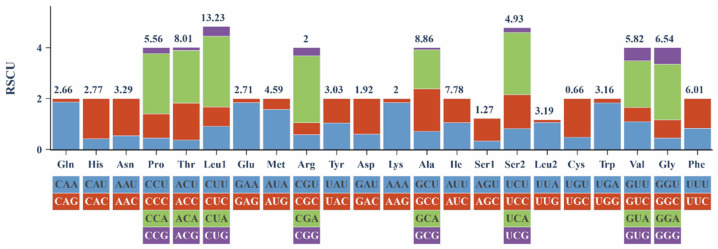
The relative synonymous codon usage (RSCU) of *P. rabaudi* mitochondrial genome.

The relative synonymous codon usage (RSCU) values for the 13 PCGs in the mitochondrial genome are exhibited in Figure 3. Leu1 (13.23%), Ala (8.86%), and Thr (8.01%) were the most frequently encoded amino acids, while Cys (0.66%) showed the lowest frequency. At the synonymous codon level, CUA (Leu1), ACA (Thr), AUU (Ile), GCC (Ala), and AUC (Ile) were the five most commonly used codons.

### 3.4. Phylogenetic Analysis

**Figure 4:**
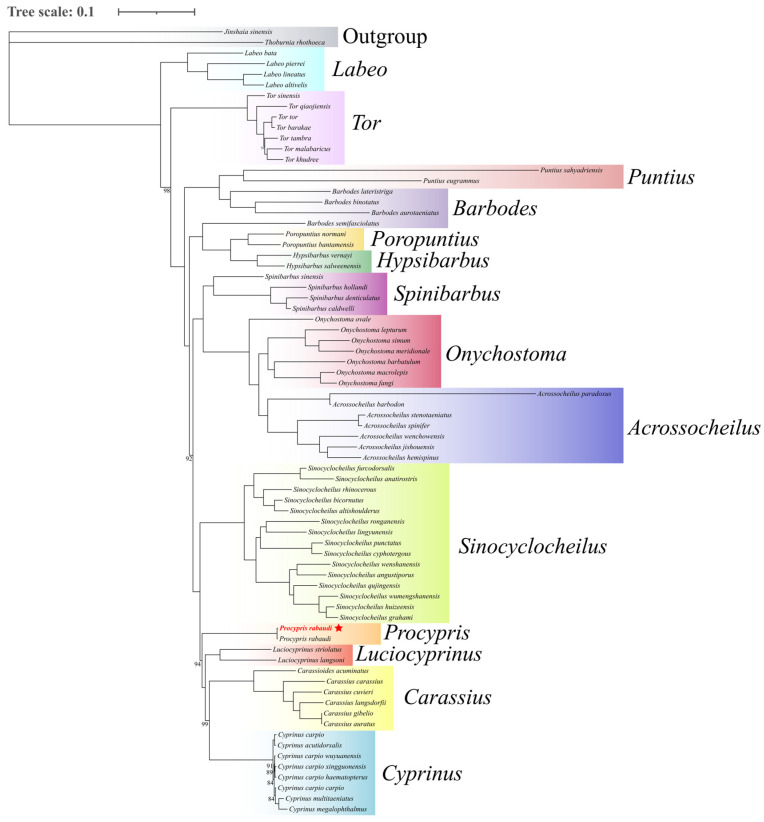
The BI phylogenetic tree of 74 cyprinid species using Bayesian inference based on 13 PCGs (The red species marked with an asterisk are the subjects of this study).

This study employed 13 PCGs to reconstruct the phylogeny of 74 cyprinid species using Bayesian inference, with *J. sinensis* and *T. rhothoeca* designated as outgroups. The results demonstrated robust nodal support values, indicating a high reliability of the phylogenetic hypotheses (Figure 4). Molecular phylogenetic analyses unequivocally placed *P. rabaudi* as a monophyletic lineage within the genus *Procypris*. The genus *Procypris* formed a strongly supported sister relationship with genus *Luciocyprinus* and, together with *Cyprinus*, *Carassioides*, and *Carassius*, constituted a well-defined monophyletic clade. Notably, the genus *Sinocyclocheilus* exhibited distinct monophyly and, along with the aforementioned clade, formed the tribe Cyprinini. Furthermore, *Acrossocheilus* and *Onychostoma* clustered together as an independent evolutionary lineage, establishing the distinct tribe Acrossocheilini. *Spinibarbus* emerged as a separate lineage designated as the tribe Spinibarbini, which maintained a stable sister-group relationship with Acrossocheilini. These findings provide novel molecular evidence for cyprinid systematics, not only validating the monophyly of certain traditionally recognized groups but also offering significant insights for further resolving phylogenetic relationships within Cyprinidae.

### 3.5. Population Size Dynamics of P. rabaudi

**Figure 5:**
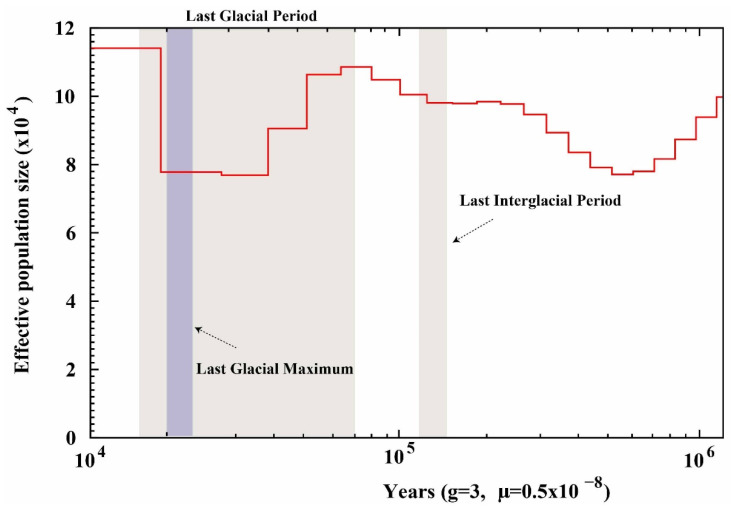
The population dynamics of *P. rabaudi* inferred from the PSMC model, the red line represents the fluctuation trend of its population size.

The population dynamics of *P. rabaudi* inferred from the PSMC model are illustrated in Figure 5. The results indicate a pronounced population expansion prior to the Last Interglacial Period. During the Last Glacial Period, the N_e_ reached a peak, followed by a prolonged decline. Notably, a secondary increase in N_e_ occurred during the late Last Glacial Period, exhibiting a bimodal population dynamic pattern. These fluctuations likely reflect the species’ adaptive responses to cyclical climatic changes.

## 4. Discussion

The whole-genome survey of *P. rabaudi* revealed several distinctive genomic features that clarify its evolutionary position within the family Cyprinidae. High sequencing quality was demonstrated by Q20 and Q30 scores of 99.61% and 98.49%, respectively, indicating excellent base-calling accuracy. The estimated genome size of 1.50 Gb falls within the known range for cyprinids, comparable to *Cyprinus carpio* (1.70 Gb) [31], *Carassius auratus* (1.73 Gb) [32], *Acrossocheilus fasciatus* (879.52 Mb) [33], and *Parabramis pekinensis* (1.03 Gb) [34]. The genome of the rock carp shows tetraploid characteristics (AABB type accounts for 74%), and the size of its genome is approximately twice that of other diploid cyprinidae fish, and is comparable to that of tetraploid cyprinidae fish, which is also evidence that the Iwahara carp is tetraploid. The genomic structure revealed by the survey analysis based on the second-generation sequencing data in this study is basically consistent with the published genome of the rock carp [28]. In addition, this study further analyzed the heterozygosity of the genome, phylogenetic relationships, and population historical dynamics. The genome heterozygosity rate of 0.44%, which is below 0.5%, suggests a relatively simple genome structure thatmay be related to the relatively low genetic diversity of artificially bred populations; inbreeding during artificial breeding can lead to a decrease in genomic heterozygosity and genetic diversity of farmed species [35], and this phenomenon has been confirmed in aquaculture salmon [36]. This result is consistent with previous studies based on microsatellite or mitochondrial markers [4,5,6], indicating that the genetic diversity of wild populations of rock carp has shown a downward trend under the influence of long-term fishing pressure and habitat fragmentation; this suggests that we need to pay attention to the impact of artificial intervention measures on protecting the genetic diversity of rare species. Moreover, the high repeat content (61.47%) of *P. rabaudi* genome exceeds that of most other cyprinids, such as *Barbodes wynaadensis* (48.26%) [37], *Schizothorax macropogon* (49.61%) [38], and *Plagiognathops microlepis* (51.18%) [39]. High repetitive sequence content is usually associated with genomic expansion, transposition element explosion or weakened selection pressure on repetitive sequences [40]. In vertebrates, the expansion of repetitive sequences, especially transposition elements, is often associated with species adaptive evolution, chromosomal rearrangement and the formation of new genes [41]. As a benthic fish with specialized distribution in the upper reaches of the Yangtze River, the high repetition sequence of the rock carp may be related to its adaptive evolution to special habitats such as rapids and low temperatures, or it may be the result of genetic drift after being in a small population state for a long time. These likely account for its larger genome size and reflect either a burst of transposable elements or relaxed selection against repeats [42] and provides important insights for the research on the adaptive evolution of *P. rabaudi*.

In aquaculture genetics, microsatellite markers are prized for high polymorphism and ease of detection, which are essential for strain identification, broodstock tracking, and quantitative trait locus (QTL) mapping related to growth and disease resistance [43,44]. The advent of high-throughput sequencing has expedited the discovery of microsatellites at a reduced cost, resulting in extensive candidate sets that enhance precision breeding and resource management [15,45]. In our study, we identified 1,151,980 SSR loci within the *P. rabaudi* genome, yielding an impressive density of 770.3 loci/Mb. This surpasses the densities observed in *C. carpio* (621.95 loci/Mb) [46] and *Hypophthalmichthys molitrix* (437.73 loci/Mb) [47]. In terms of SSR type distribution, single-nucleotide repeats are dominant (55.34%), among which A/T motifs are the most abundant. This is related to the high content of AT in the genomes of most fish and the fact that the replication slip mechanism dominates the formation of microsatellites [14,47,48]. The dinucleotide repeats are mainly AC/GT, while the trinucleotide repeats are mostly AAT/ATT. The distribution characteristics of these motifs are similar to those of other species in the family Cyprinidae [46]. It is worth noting that trinucleotide and above repeats in the gene coding region are often associated with functional variations, which may affect gene expression or protein structure [49,50]. Therefore, the SSR loci identified in this study can not only be used in population genetics research, but also further screen for functional microsatellites related to traits such as growth, disease resistance and adaptation in the future, providing a molecular basis for the breeding of superior varieties of the rock carp.

The complete mitochondrial genome size of *P. rabaudi* was 16,595 bp, aligning with previous reports [51]. It comprises the canonical 13 PCGs, 22 tRNAs, two rRNAs, and the control region, similar to mitochondrial architecture across Cyprinidae [52,53]. Most genes initiate with ATG, but *COXI* initiates with GTG. This GTG start is a typical cypriniform trait also seen in *Luciocyprinus langsoni* and *Linichthys laticeps* [53,54]. The mitochondrial genome of *P. rabaudi* contains incomplete stop codons (TA and T), which are commonly observed in fish mitochondrial genomes. These incomplete codons likely undergo post-transcriptional polyadenylation to form complete termini [54]. Codon usage preference analysis revealed that Leu1 had the highest usage frequency (13.23%) and Cys had the lowest (0.66%) in the mitochondrial genome of the rock carp. Leucine, as a hydrophobic amino acid, plays a significant role in the composition of mitochondrial membrane proteins and respiratory chain complexes [55]. Its high usage frequency may be related to the adaptation of mitochondrial metabolic efficiency, previous studies have shown that leucine can regulate mitochondrial function through the mTOR and Opa-1 signaling pathways, suggesting its potential role in energy metabolism adaptation [56].

In this study, a phylogenetic tree was constructed based on 13 mitochondrial PCGs. The results indicated that the genus *Procypris* was monophyletic and formed a stable sister group relationship with the genus *Luciocyprinus*. Notably, previous studies have suggested a closer phylogenetic relationship between *Procypris* and the genus *Sinocyclocheilus* [54,57]. This discrepancy may be attributed to differences in sample selection and the models used for tree construction. There is some debate regarding the close relationship of *Procypris* with Cyprininae and Barbinae. Liu et al. found that *P. rabaudi* was more closely related to *Barbinae* using the RAPD method [58]. In contrast, Li et al. and Zhang et al. indicated that genus *Procypris* had a closer phylogenetic relationship with Cyprininae than with Barbinae [51,52]. In this study, the genus *Procypris*, along with the genera *Cyprinus*, *Carassioides*, and *Carassius*, formed a highly supported monophyletic group, supporting the view that *Procypris* belongs to the subfamily Cyprininae. These results provide robust molecular evidence for the evolutionary relationships of *Procypris* species, and future research should incorporate nuclear genes and additional species to clarify the taxonomic status of the family Cyprinidae.

Studying the historical population dynamics of fish can reveal the impacts of climate change and fluctuations in water environments on fish population sizes, providing a scientific basis for predicting future ecological responses and formulating conservation management strategies [59,60]. Our PSMC analysis indicates that *P. rabaudi* experienced significant population expansion prior to the Last Interglacial Period, which is closely related to the climate warming at that time that led to the expansion of suitable habitats [61]. During the subsequent Last Glacial Period, the effective population size of *P. rabaudi* showed a continuous declining trend, likely due to the sharp drop in temperature causing habitat shrinkage and fragmentation, along with deteriorated hydrological conditions that limited population connectivity, thereby affecting reproductive success and genetic diversity [62,63]. It is worth noting that during the last glacial period, the rock carp exhibited bimodal population dynamics; that is, a brief secondary growth occurred within the overall downward trend. This fluctuation may be related to the periodic warming of the climate or the existence of local shelters during the ice age; the previous study of the *Schizothorax prenanti* [64] and *Schizothorax* species complex in Yunnan [65] supported this view, with our results also suggesting that the rock carp has a certain population recovery and adaptability in historical climate fluctuations. Similar climate-driven population fluctuations have been reported in various cyprinidae species, indicating that the Quaternary climate cycle has a profound impact on the distribution and genetic structure of freshwater fish in East Asia [66]. As a species endemic to the upper reaches of the Yangtze River, the population historical dynamics of the rock carp not only reflect the regional response to global climate change, but also reveal the habitat reconstruction process of river ecosystems during the glacial and interglacial cycles. These historical fluctuations have significant impacts on the genetic diversity, adaptation potential and extinction risk of the current population, suggesting that special attention should be paid to its vulnerability to climate change in future conservation efforts. This response pattern has been recorded in cyprinid fish, reflecting the profound impact of periodic climate changes on the evolutionary history of cyprinids. These findings provide important guidance for formulating effective conservation strategies for *P. rabaudi*.

## 5. Conclusions

This study provides the whole-genome survey of the endangered *P. rabaudi*. Key genomic features include a size of ~1.50 Gb, 0.44% heterozygosity, and a high repeat content of 61.47%. We identified 1,151,980 SSR loci, predominantly mononucleotide repeats, offering valuable markers for future population genetics and breeding. The complete mitogenome (16,595 bp) was assembled. Phylogenetic analysis based on 13 mitochondrial PCGs robustly places *Procypris* as sister to *Luciocyprinus*, and together they cluster with *Cyprinus*, *Carassioides*, and *Carassius* within Cyprininae. PSMC analysis revealed significant pre-Last Interglacial population expansion and a peak during the Last Glacial Period, followed by a decline with a bimodal fluctuation pattern, reflecting responses to Pleistocene climatic shifts. These genomic resources and insights form a crucial foundation for the conservation and genetic management of *P. rabaudi* and enhance our understanding of cyprinid phylogeny and evolution.

## Figures and Tables

**Table 1:** The library sequencing statistics of *P. rabaudi*.

Type	Reads Number	Base Count (Gb)	Read Length (bp)	Q20 (%)	Q30 (%)	GC Content (%)
raw	1,112,770,540	166.92	150	99.61	98.50	38.91
dedup	1,069,509,006	157.79	147	99.61	98.49	38.65

**Table 2:** Summary of *P. rabaudi* genomic features based on K-mer analysis (K-mer = 17).

K-mer Number	K-mer Depth	Genome Size (bp)	Revised Genome Size (bp)	Heterozygous Ratio (%)	Repeat (%)
140,489,127,949	92	1,495,420,000	1,490,725,446	0.44	61.47

**Table 3:** The draft genome assembly result of *P. rabaudi*.

Assembly Level	Total Number	Total Base (bp)	Max Length (bp)	N50 Length (bp)	N90 Length (bp)
Scaffold	1,017,551	1,881,216,104	77,646	7176	1292
Contig	4,385,755	1,368,591,351	10,405	469	129

**Table 4:** Statistics of microsatellite identification results.

Assembly Level	N90 Length (bp)
Total number of sequences examined	1,017,551
Total size of examined sequences (bp)	1,881,216,104
Total number of identified SSRs	1,151,980
Number of SSR containing sequences	398,918
Number of sequences containing more than 1 SSR	227,348
Number of SSRs present in compound formation	156,448

## Data Availability

The next-generation sequencing data based BGI platform have been uploaded in to the NCBI SRA database with access number SRR31066341 (https://identifiers.org/ncbi/insdc.sra:SRR31066341). The assembled draft genome based on next-generation sequencing data can be obtained by contacting the corresponding author. All Supplementary Materials are uploaded as attachments and are available.

## Supplementary Materials

The following supporting information can be downloaded at: https://www.mdpi.com/article/10.3390/ani16020246/s1, Table S1: GenBank accession numbers of mitogenomes of 74 species used in this study; Table S2: Mitochondrial genome composition and characteristics of *P. rabaudi*.
