# Supplementary material for: Genomic Characterization and Phylogenetic Relationships of Procypris rabaudi Revealed by Whole-Genome Survey Analysis
Source: Animals (Basel). 2026 Jan 14;16(2):246. doi: 10.3390/ani16020246 (PMC12837989; doi:10.3390/ani16020246)
Supplement: Supplementary file 1 [file animals-16-00246-s001.zip › Table S2.pdf]

Table S2. Mitochondrial genome composition and characteristics of *P. rabaudi.*

Gene	Position-start	Position-end	Length (bp)	Amino acid	Start codon	Stop codon	Strand
tRNA-Phe	1	69	69				H
12S RNA	70	1027	958				H
tRNA-Val	1028	1099	72				H
16S RNA	1100	2782	1683				H
tRNA-Leu	2783	2858	76				H
ND1	2860	3834	975	324	ATG	TAA	H
tRNA-Ile	3839	3910	72				H
tRNA-Gln	3909	3979	71				L
tRNA-Met	3981	4049	69				H
ND2	4050	5094	1045	349	ATG	T	H
tRNA-Trp	5095	5165	71				H
tRNA-Ala	5168	5236	69				L
tRNA-Asn	5238	5310	73				L
tRNA-Cys	5343	5409	67				L
tRNA-Tyr	5409	5479	71				L
COXI	5481	7031	1551	516	GTG	TAA	H
tRNA-Ser	7032	7102	71				L
tRNA-Asp	7106	7177	72				H
COXII	7183	7873	691	231	ATG	T	H
tRNA-Lys	7874	7950	77				H
ATPase8	7952	8116	165	54	ATG	TAG	H
ATPase6	8110	8792	683	228	ATG	TA	H
COXIII	8793	9577	785	262	ATG	TA	H
tRNA-Gly	9578	9649	72				H
ND3	9650	9998	349	117	ATG	T	H
tRNA-Arg	9999	10068	70				H
ND4L	10069	10365	297	98	ATG	TAA	H
ND4	10359	11739	1381	461	ATG	T	H
tRNA-His	11740	11808	69				H
tRNA-Ser	11809	11877	69				H
tRNA-Leu	11879	11951	73				H
ND5	11955	13778	1824	607	ATG	TAA	H
ND6	13775	14296	522	173	ATG	TAA	L
tRNA-Glu	14297	14365	69				L
Cytb	14371	15511	1141	381	ATG	T	H
tRNA-Thr	15512	15583	72				H
tRNA-Pro	15583	15652	70				L
D-loop	15653	16595	943				H
