# Supplementary material for: Genomic Characterization and Phylogenetic Relationships of Procypris rabaudi Revealed by Whole-Genome Survey Analysis
Source: Animals (Basel). 2026 Jan 14;16(2):246. doi: 10.3390/ani16020246 (PMC12837989; doi:10.3390/ani16020246)
Supplement: Supplementary file 1 [file animals-16-00246-s001.zip › Table S1.pdf]

Table S1 GenBank accession numbers of mitogenomes of 74 species used in this study.

Genus	Species	Length (bp)	GenBank Accession Number
Acrossocheilus	*Acrossocheilus barbodon*	16596	NC_022184
	Acrossocheilus hemispinus	16590	NC_022183
	Acrossocheilus jishouensis	16587	NC_034917.1
	Acrossocheilus paradoxus	16597	NC_029453
	Acrossocheilus spinifer	16591	NC_034918.1
	Acrossocheilus stenotaeniatus	16594	NC_024934
	Acrossocheilus wenchowensis	16591	NC_020145.1
Barbodes	*Barbodes aurotaeniatus*	16562	NC_031619
	Barbodes binotatus	16573	NC_034755.1
	Barbodes lateristriga	16586	NC_031588
	Barbodes semifasciolatus	16594	NC_020096.1
Carassioides	*Carassioides acuminatus*	16579	NC_031430
Carassius	*Carassius auratus*	16581	KM659025.1
	Carassius carassius	16597	JQ911695.1
	Carassius cuvieri	16581	NC_010768.1
	Carassius gibelio	16581	NC_014177.1
	Carassius langsdorfii	16578	NC_002079.1
Cyprinus	*Cyprinus acutidorsalis*	16581	OQ871460.1
	Cyprinus carpio	16581	OL699932.1
	Cyprinus carpio carpio	16581	NC_018035.1
	Cyprinus carpio haematopterus	16581	NC_018037.1
	Cyprinus carpio wuyuanensis	16582	NC_018039.1
	Cyprinus carpio xingguonensis	16581	NC_018036.1
	Cyprinus megalophthalmus	16580	NC_028417.1
	Cyprinus multitaeniatus	16580	NC_028419.1
Hypsibarbus	*Hypsibarbus salweenensis*	16588	NC_031620
	Hypsibarbus vernayi	16590	NC_031621
Jinshaia	*Jinshaia sinensis*	16567	NC_024598
Labeo	*Labeo altivelis*	16603	NC_029444
	Labeo bata	16605	NC_015193.1
	Labeo lineatus	16606	NC_022956
	Labeo pierrei	16766	NC_022943
Luciocyprinus	*Luciocyprinus langsoni*	16586	NC_066657.1
	Luciocyprinus striolatus	16601	NC_029436
Onychostoma	*Onychostoma barbatulum*	16597	NC_021644.1
	Onychostoma fangi	16597	NC_031529
	Onychostoma lepturum	16601	NC_054158.1
	Onychostoma macrolepis	16595	NC_023799
	Onychostoma meridionale	16595	NC_031603
	Onychostoma ovale	16602	NC_066040.1
	Onychostoma simum	16601	NC_021972.1

Poropuntius	*Poropuntius bantamensis*	16594	NC_031604
	Poropuntius normani	16592	NC_031589
Procypris	*Procypris rabaudi*	16595	NC_011192.1
Puntius	*Puntius eugrammus*	16847	NC_031611
	Puntius sahyadriensis	16798	NC_033916.1
Sinocyclocheilus	*Sinocyclocheilus altishoulderus*	16589	NC_013186
	Sinocyclocheilus anatirostris	16577	NC_069226.1
	Sinocyclocheilus angustiporus	16587	NC_060848.1
	Sinocyclocheilus bicornutus	17426	NC_031382
	Sinocyclocheilus cyphotergous	16611	NC_072977.1
	Sinocyclocheilus furcodorsalis	16581	NC_019995.1
	Sinocyclocheilus grahami	16585	NC_013189
	Sinocyclocheilus huizeensis	16585	NC_044072.1
	Sinocyclocheilus lingyunensis	16572	NC_056143.1
	Sinocyclocheilus punctatus	16582	NC_058003.1
	Sinocyclocheilus qujingensis	16588	NC_043910.1
	Sinocyclocheilus rhinocerous	16588	NC_027168
	Sinocyclocheilus ronganensis	16587	NC_032385
	Sinocyclocheilus wenshanensis	16595	NC_060737.1
	Sinocyclocheilus wumengshanensis	16585	NC_039769.1
Spinibarbus	*Spinibarbus caldwelli*	16545	NC_022149
	Spinibarbus denticulatus	16549	NC_021616
	Spinibarbus hollandi	16521	NC_026129
	Spinibarbus sinensis	16591	NC_022465
Thoburnia	*Thoburnia rhothoeca*	16626	NC_031633
Tor	*Tor barakae*	16780	NC_056296.1
	Tor khudree	16573	NC_027617
	Tor malabaricus	16580	NC_036383.1
	Tor qiaojiensis	16590	NC_066814.1
	Tor sinensis	16579	NC_022702.1
	Tor tambra	16581	NC_036511.1
	Tor tor	16554	NC_027498
